# Data for “joint modeling of lithosphere and mantle dynamics: Sensitivity to viscosities within the lithosphere, asthenosphere, transition zone, and D" layers”

**DOI:** 10.1016/j.dib.2019.104935

**Published:** 2019-12-05

**Authors:** Xinguo Wang, William E. Holt, Attreyee Ghosh

**Affiliations:** a Key Laboratory of Continental Collision and Plateau Uplift, Institute of Tibetan Plateau Research, Chinese Academy of Sciences, Beijing, China; b State Key Laboratory of Earthquake Dynamics, Institute of Geology, China Earthquake Administration, Beijing, China; c Department of Geosciences, Stony Brook University, Stony Brook, NY, USA; d Centre for Earth Sciences, Indian Institute of Science, Bangalore, India

**Keywords:** Mantle viscosity, Geoid, Plate motion, Strain rate, Stress

## Abstract

The article presents the data calculated from four different viscosity structures V1, V2 [1], SH08 [2], and GHW13 [3], as well as two tomography models S40RTS [4] and SAW642AN [5], using the joint modeling of lithosphere and mantle dynamics technique [3, 6–9]. Besides, the data contain the information on the viscosity variations of the lithosphere, asthenosphere, transition zone, and D″ layer based on the viscosity structure SH08.

**Table:** Specifications Table

Subject	Earth and Planetary Sciences
Specific subject area	Mantle viscosity
Type of data	Table Figure
How data were acquired	Data are output from approach of the joint modeling of lithosphere and mantle dynamics
Data format	Analyzed
Parameters for data collection	The lithosphere gravitational potential energy differences, lateral viscosity variations of the lithosphere, the horizontal tractions from mantle convection, viscosity structures, and tomography models
Description of data collection	The horizontal tractions and geoid are calculated by HC, the lithosphere stresses, plate motions, and strain rates are computed from the lithosphere finite element model
Data source location	Tomography model S40RTS, University of Michigan, Ann Arbor, U.S.A Tomography model SAW642AN, University of California, Berkeley, U.S.A Viscosity structures V1, V2, University of Toronto, Toronto, Canada Viscosity structure SH08, Geological Survey of Norway, Trondheim, Norway Viscosity structure GHW13, Stony Brook University, Stony Brook, U.S.A
Data accessibility	With the article
Related research article	Xinguo Wang, William E. Holt, Attreyee Ghosh Physics of the Earth and Planetary Interiors https://doi.org/10.1016/j.pepi.2019.05.006

**Table:** 

**Value of the Data** • The data provide quantitative information on the global geoid, stresses, plate motions, and strain rates between the surface observables and predications from the four viscosity structures, the viscosity variations within the lithosphere, asthenosphere, transition zone, and D″ layer, as well as two global tomography models. • The data explore the effects of radial viscosity variations, within the four layers of the lithosphere, asthenosphere, transition zone, and D″ layer, on lithosphere deformation, which are valuable for understanding tectonic forces. • The model and data can be used to refine the mantle viscosity and mantle convection patterns based on new high-resolution global seismic tomography models.

## 1 Data

Here, we present the data computed from the joint modeling of lithosphere and mantle dynamics technique. Four tables contain the information on the sensitivities to the variations of the viscosities within the lithosphere, asthenosphere, transition zone, and D″ layers. Four figures show the computed stresses, plate velocities, and strain rates, along with the observables based on the tomography model S40RTS [4] and the viscosity structure SH08 [2].

## 2 Experimental design, materials, and methods

**Fig. 1:**
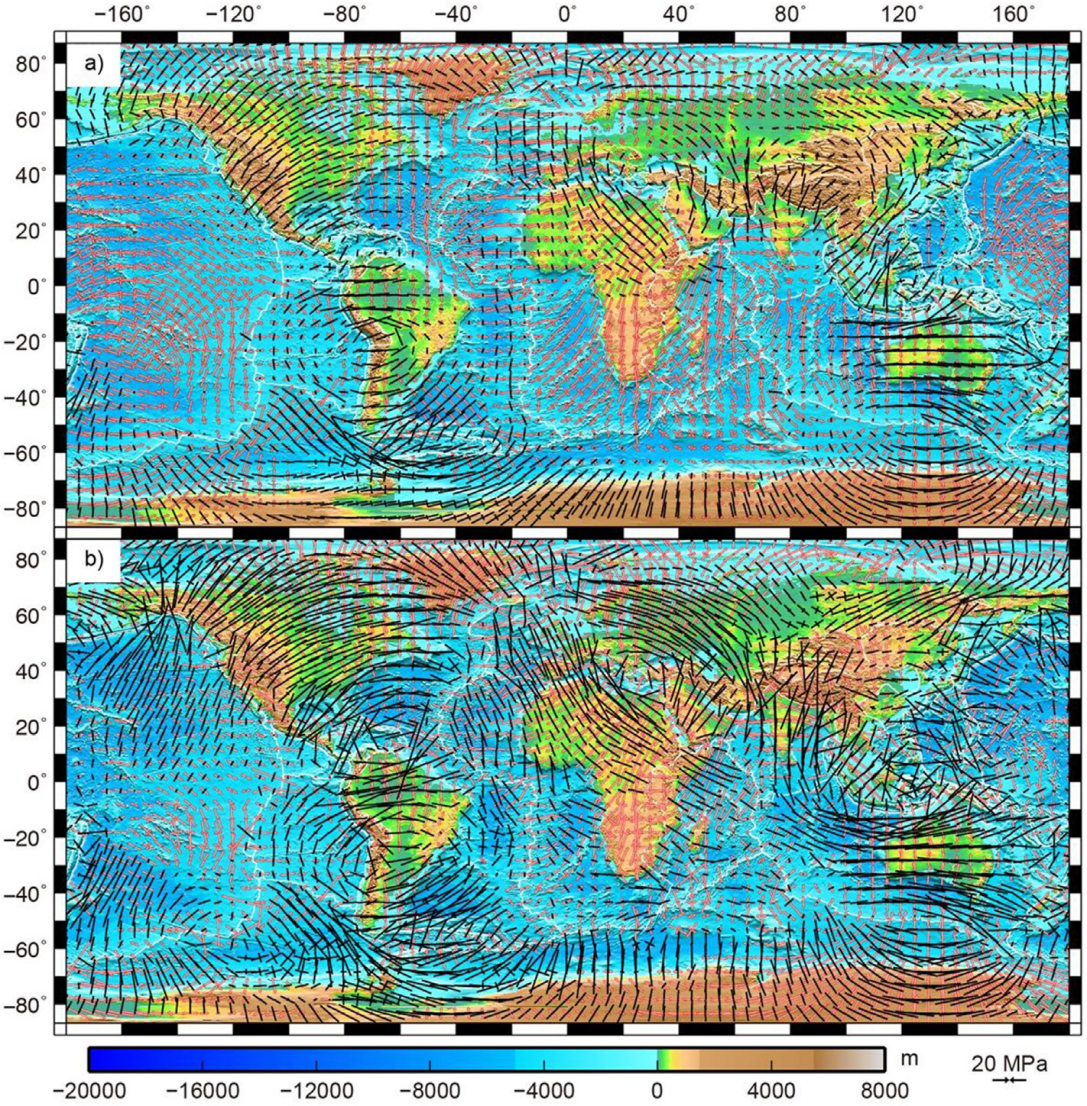
Global deviatoric stresses resulting from the tractions, plotted at every 5° on top of ETOPO1 topography. The traction related stresses are calculated from mantle flows based on the seismic tomography model S40RTS [4] and the radial viscosity structure SH08 [2]. Tensional deviatoric stresses are shown by red arrows, while compressional deviatoric stresses are shown by black arrows. Strike-slip regions are indicated by one tensional and one compressional pair of arrows. Length of the arrows is proportional to the magnitude of vertically integrated stresses. (b) The combined deviatoric stresses from both the traction related stresses and GPE differences. The GPE differences are given by Ghosh et al. (2013) [3].

**Fig. 2:**
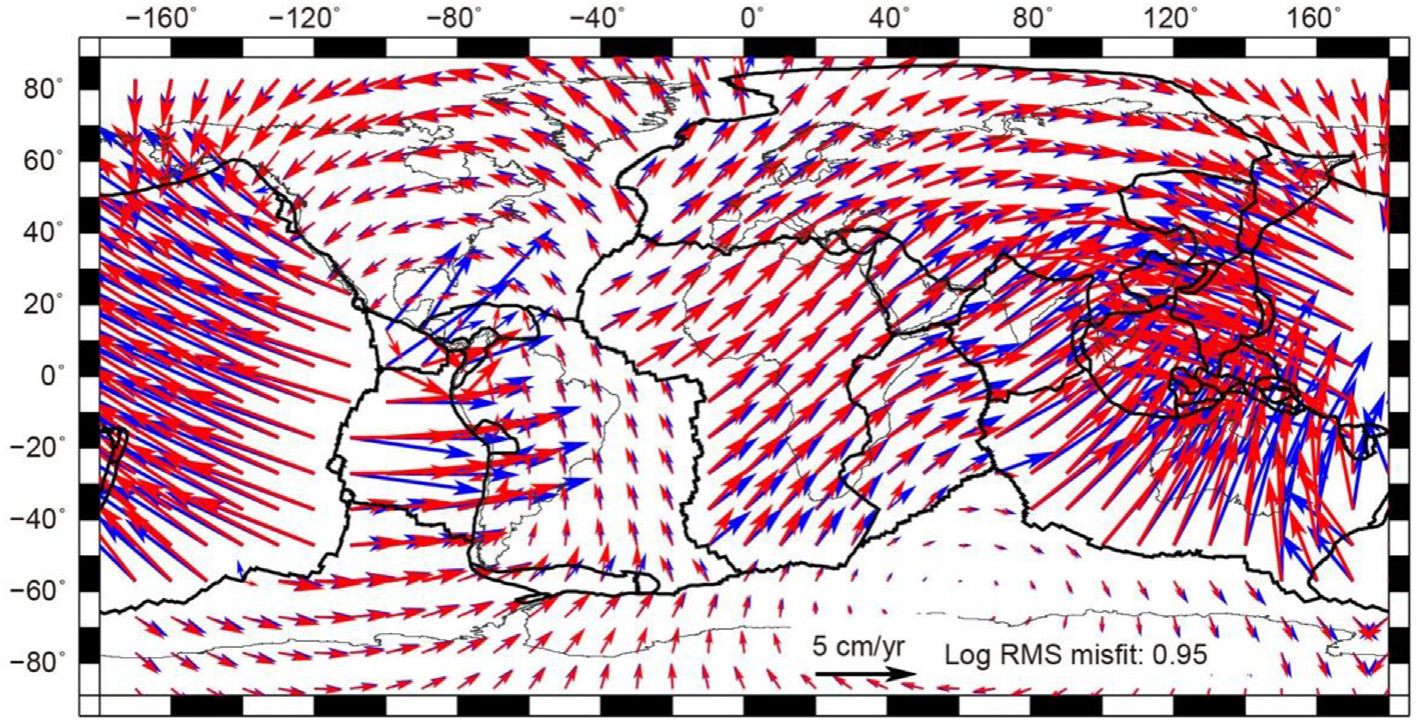
Kinematic no-net-rotation (NNR) model from Kreemer et al. (2006) [13] (blue arrows) along with the predicted velocities in an NNR frame (red arrows) from our global dynamic model. The legend for the velocity and global log of RMS misfit are noted on the center bottom. The predicted velocities are computed from the combined stresses (Fig. 1b).

**Fig. 3:**
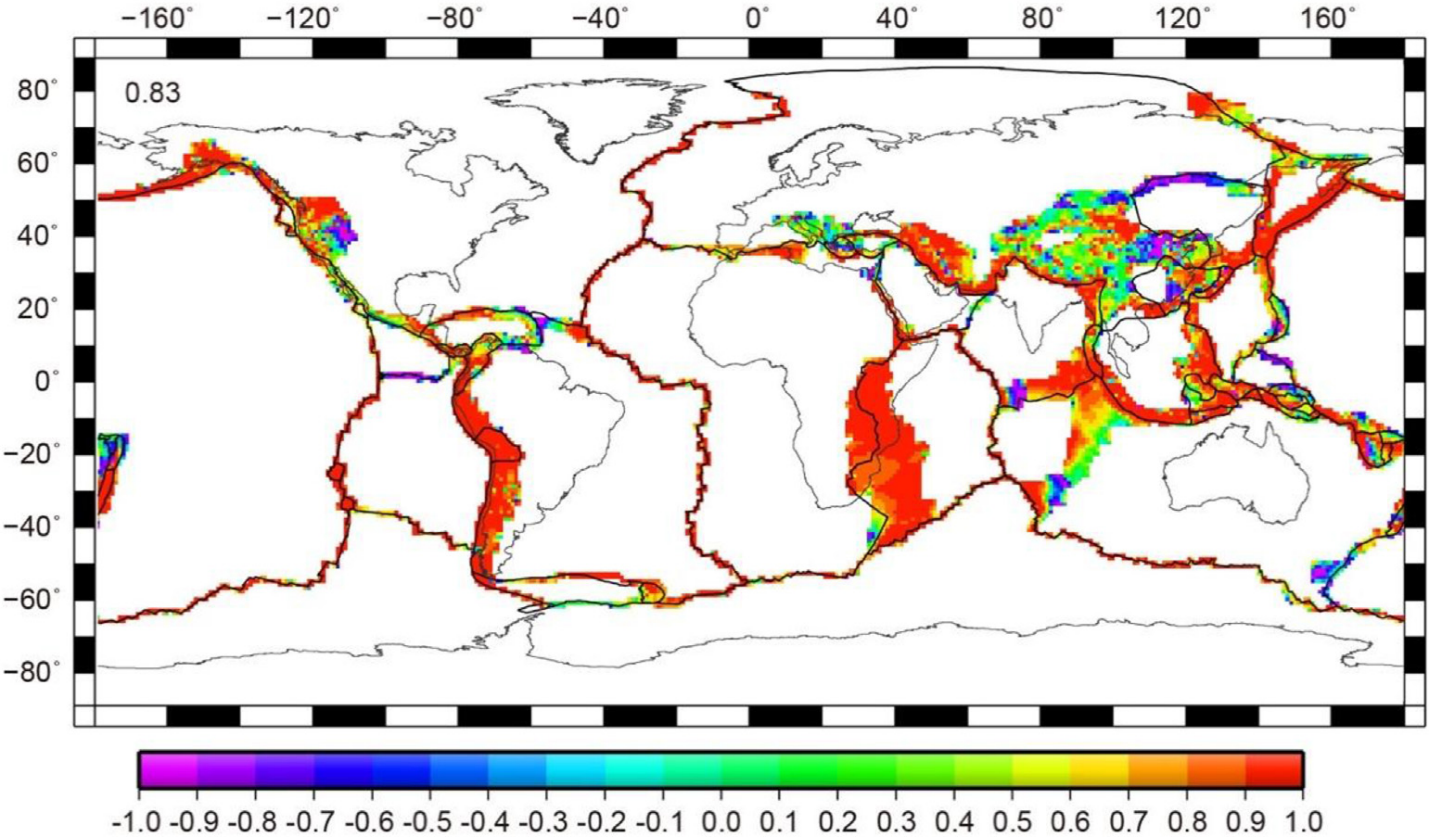
Correlation coefficients between the deviatoric stress tensors from the combined stresses (Fig. 1b) and the Global Strain Rate Map [12]. The correlation coefficient is noted on top left.

We use the joint lithosphere and mantle dynamics modeling technique to calculate the lithosphere deviatoric stresses, plate motions, and strain rates [3,[6], [7], [8], [9]]. This approach needs the lithosphere gravitational potential energy (GPE) differences and the horizontal tractions as inputs to the lithosphere finite element model. The GPE gradients and the lateral lithosphere viscosity structure are provided by Ghosh et al. (2013) [3], which are obtained from their optimal model. The tractions and predicated geoid are calculated from HC [10,11]. We calculate the deviatoric stresses (Fig. 1), plate motions (Fig. 2), strain rates (Fig. 3), and geoid based on the tomography models S40RTS [4] and SAW642AN [5] and the viscosity structure SH08 [2].

To quantify the sensitivities of the viscosities within the lithosphere, asthenosphere, transition zone, and D″ layer, we compare the computed deviatoric stresses, plate motions, strain rates, and geoid with the surface observations, such as, World Stress Map (WSM) [[16], [17], [18]], Global Strain Rate Map (GSRM) strain rate tensors [12], surface motions in a no-net-rotation (NNR) frame with the velocities of Kreemer et al. (2006) [13], and the observed geoid from Chambat et al. (2010) [14], respectively. The detailed comparison methods are shown as following.

We follow the equation provided by Flesch et al. (2007) [15] to compute correlation coefficients (hereby named GSRM in Table 1, Table 2, Table 3, Table 4) between the deviatoric stress tensors and the GSRM strain rate tensors, (hereby referred to as $Cor_{GSRM}$). The equation is:

(1) $$-1\leq \sum_{areas}\left(\varepsilon •\tau \right)\Delta S/\left(\sqrt{\underset{areas}{\sum }\left(E^{2}\right)\Delta S}*\sqrt{\underset{areas}{\sum }\left(T^{2}\right)\Delta S}\right)\leq 1\;$$

where $E=\sqrt{\varepsilon_{\phi \phi }^{2}+\varepsilon_{\theta \theta }^{2}+\varepsilon_{\gamma \gamma }^{2}+\varepsilon_{\phi \theta }^{2}+\varepsilon_{\theta \phi }^{2}}=\sqrt{2\varepsilon_{\phi \phi }^{2}+2\varepsilon_{\phi \phi }\varepsilon_{\theta \theta }+2\varepsilon_{\theta \theta }^{2}+2\varepsilon_{\phi \theta }^{2}}$, $T=\sqrt{\tau_{\phi \phi }^{2}+\tau_{\theta \theta }^{2}+\tau_{\gamma \gamma }^{2}+\tau_{\phi \theta }^{2}+\tau_{\theta \phi }^{2}}=\sqrt{2\tau_{\phi \phi }^{2}+2\tau_{\phi \phi }\tau_{\theta \theta }+2\tau_{\theta \theta }^{2}+2\tau_{\phi \theta }^{2}}$ and $\varepsilon •\tau =2\varepsilon_{\phi \phi }\tau_{\phi \phi }+\varepsilon_{\phi \phi }\tau_{\theta \theta }+\varepsilon_{\theta \theta }\tau_{\phi \phi }+2\varepsilon_{\theta \theta }\tau_{\theta \theta }+2\varepsilon_{\phi \theta }\tau_{\phi \theta }$.

**Fig. 4:**
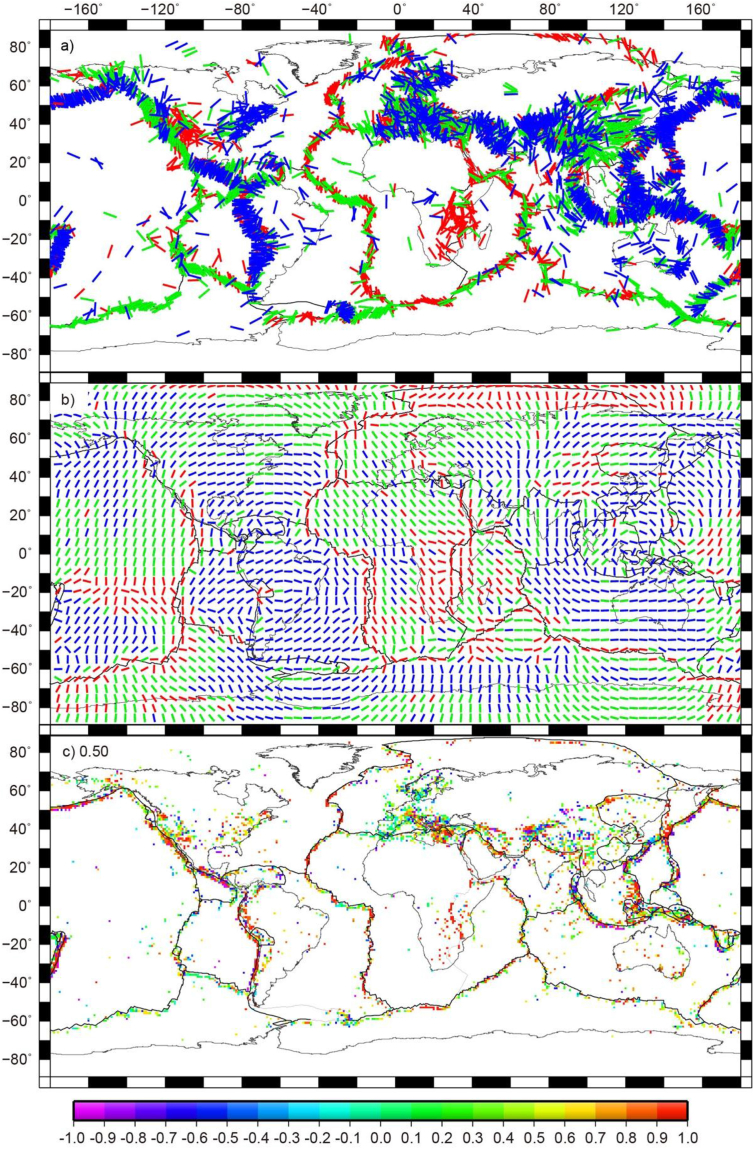
(a) *SH*_max_ directions (maximum horizontal stress orientations) from the World Stress Map [[16], [17], [18]] averaged within 1° × 1° areas. Red indicates normal fault regime, blue indicates thrust regime, whereas green denotes strike-slip regime. (b) The most compressive principal axes of the stress tensors from the combined stresses (Fig. 1b). The colors indicate the strain environment predicted by the deviatoric stresses of the model. Red indicates the maximum horizontal compression orientation in a normal fault regime, blue indicates the maximum horizontal compression in a thrust fault regime, and green denotes the maximum horizontal compressive stress direction in a strike-slip regime. (c) Correlation coefficients between the predicted stress tensors and from the World Stress Map stresses.

Here *E* and *T* represent the second invariants of strain rate and stress, respectively. $\varepsilon_{ij}$ are the strain rates from the GSRM [12], $\tau_{ij}$ are the computed deviatoric stress tensors from the combined stresses, and ΔS is the grid area. We also use equation (1) to calculate the correlation coefficient (hereby named WSM in Table 1 and Fig. 4) between the most compressive principal axes directions and styles (Fig. 4b) from the combined stresses and the WSM [[16], [17], [18]]. In this case, *E* and $\varepsilon_{ij}$ in equation (1) are computed from WSM.

**Table 1:** The global geoid and Global Strain Rate Map (GSRM) correlation coefficients, log of RMS misfit (surface motions in mm/yr), and total errors ($\varepsilon_{total\_error}$). These correlation coefficients for the geoid and GSRM, RMS misfits are calculated between the surface observables [[12], [13], [14]] and predictions from the seismic tomography models S40RTS [4] and SAW642AN [5], as well as the viscosity structures V1, V2, SH08, and GHW13 [[1], [2], [3]].

Tomography	Items	Viscosities									
Models		GHW13	V1	V1_LM	V1_MM	V1_SM	V2	V2_LM	V2_MM	V2_SM	SH08
S40RTS	VRMS	97.39	68.60	42.25	26.18	40.37	71.74	30.74	27.22	57.46	33.99
	Geoid	0.36	0.50	0.78	0.89	0.74	0.44	0.77	0.82	0.53	0.82
	GSRM	0.86	0.56	0.85	0.83	0.78	0.50	0.85	0.82	0.67	0.83
	log RMS	0.98	1.33	1.05	1.01	1.06	1.37	0.93	0.95	1.21	0.95
	Total errors	1.76	2.27	1.42	1.29	1.54	2.43	1.31	1.31	2.01	1.30
	WSM	0.51	0.32	0.50	0.49	0.47	0.29	0.51	0.49	0.41	0.50
SAW642AN	VRMS	79.40	56.67	43.43	29.18	35.55	60.25	28.03	25.24	48.57	30.22
	Geoid	0.52	0.56	0.87	0.91	0.79	0.48	0.86	0.85	0.58	0.92
	GSRM	0.85	0.54	0.82	0.81	0.75	0.48	0.83	0.79	0.64	0.80
	log RMS	0.99	1.34	1.14	1.05	1.07	1.38	0.97	0.96	1.22	0.98
	Total errors	1.62	2.24	1.45	1.33	1.53	2.42	1.28	1.32	2.00	1.26
	WSM	0.50	0.32	0.49	0.48	0.45	0.28	0.50	0.47	0.38	0.48

**Table 2:** Same as Table 1, except for the lithosphere versus asthenosphere viscosity embedded within the viscosity structure SH08.

Models		Aesth																													
	Litho	1.4 × 10^19^					5.4 × 10^19^					1.4 × 10^20^					5.4 × 10^20^					1.4 × 10^21^					5.4 × 10^21^				
		VRMS	geoid	GSRM	log RMS	Total errors	VRMS	geoid	GSRM	log RMS	Total errors	VRMS	geoid	GSRM	log RMS	Total errors	VRMS	geoid	GSRM	log RMS	Total errors	VRMS	geoid	GSRM	log RMS	Total errors	VRMS	geoid	GSRM	log RMS	Total errors
S40RTS	5.6 × 10^20^	134.15	0.23	0.41	1.41	2.77	97.53	0.35	0.20	1.53	2.98	83.84	0.42	0.09	1.56	3.05	73.97	0.47	−0.01	1.58	3.12	66.89	0.52	−0.09	1.59	3.16	47.46	0.68	−0.25	1.60	3.17
	8.6 × 10^20^	124.09	0.26	0.48	1.36	2.62	92.85	0.37	0.27	1.49	2.85	80.88	0.43	0.16	1.54	2.95	71.95	0.48	0.05	1.58	3.05	65.21	0.53	−0.03	1.59	3.09	46.37	0.69	−0.21	1.60	3.12
	2.6 × 10^21^	87.65	0.40	0.64	1.20	2.16	73.03	0.48	0.52	1.34	2.34	67.22	0.52	0.42	1.41	2.47	62.10	0.55	0.32	1.48	2.61	56.98	0.59	0.24	1.52	2.69	41.52	0.73	0.03	1.58	2.82
	5.6 × 10^21^	60.69	0.57	0.72	1.08	1.79	54.08	0.62	0.67	1.18	1.89	52.05	0.64	0.63	1.25	1.98	49.82	0.66	0.57	1.31	2.08	46.46	0.69	0.52	1.35	2.14	35.89	0.79	0.33	1.47	2.35
	8.6 × 10^21^	48.61	0.68	0.75	1.03	1.60	43.71	0.72	0.73	1.09	1.64	42.61	0.72	0.71	1.14	1.71	41.36	0.73	0.69	1.19	1.77	39.14	0.76	0.66	1.23	1.81	32.76	0.82	0.54	1.34	1.98
	2.6 × 10^22^	35.34	0.81	0.78	0.97	1.38	33.39	0.83	0.81	0.95	1.31	33.64	0.83	0.82	0.95	1.30	34.70	0.83	0.83	0.96	1.30	35.94	0.82	0.83	0.97	1.32	40.43	0.79	0.81	1.01	1.41
	5.6 × 10^22^	35.72	0.81	0.79	0.96	1.36	39.19	0.80	0.82	0.94	1.32	44.83	0.77	0.84	0.95	1.34	52.87	0.72	0.85	0.97	1.40	56.74	0.70	0.85	0.97	1.42	61.56	0.68	0.85	0.97	1.44
	8.6 × 10^22^	36.54	0.81	0.79	0.96	1.36	42.68	0.78	0.83	0.94	1.33	51.43	0.73	0.85	0.96	1.38	63.90	0.67	0.86	0.99	1.46	69.43	0.64	0.86	0.99	1.49	74.23	0.62	0.86	0.99	1.51
	2.6 × 10^23^	38.11	0.80	0.80	0.95	1.35	48.47	0.75	0.83	0.94	1.36	62.72	0.67	0.85	0.98	1.46	84.00	0.58	0.86	1.02	1.58	93.29	0.55	0.86	1.03	1.62	99.11	0.53	0.86	1.03	1.64
	5.6 × 10^23^	38.63	0.80	0.80	0.95	1.35	50.24	0.74	0.83	0.95	1.38	66.29	0.66	0.85	0.99	1.48	90.74	0.56	0.86	1.03	1.61	101.55	0.53	0.86	1.04	1.65	108.09	0.51	0.87	1.05	1.67
SAW642AN	5.6 × 10^20^	112.41	0.22	0.44	1.40	2.74	67.75	0.41	0.26	1.51	2.84	52.99	0.50	0.15	1.55	2.90	79.88	0.58	0.05	1.57	2.94	59.15	0.64	−0.02	1.58	2.96	36.26	0.80	−0.15	1.59	2.94
	8.6 × 10^20^	103.23	0.26	0.50	1.36	2.60	65.18	0.44	0.32	1.48	2.72	51.54	0.52	0.21	1.53	2.80	75.72	0.59	0.10	1.56	2.87	57.40	0.65	0.03	1.58	2.90	35.39	0.81	−0.12	1.59	2.90
	2.6 × 10^21^	70.80	0.47	0.64	1.20	2.09	53.36	0.58	0.52	1.34	2.24	44.47	0.63	0.43	1.42	2.36	58.37	0.67	0.33	1.48	2.48	48.90	0.72	0.26	1.52	2.54	31.50	0.85	0.07	1.57	2.65
	5.6 × 10^21^	47.96	0.69	0.70	1.08	1.69	40.59	0.74	0.66	1.19	1.79	35.58	0.76	0.61	1.26	1.89	42.46	0.78	0.55	1.33	2.00	38.47	0.81	0.49	1.38	2.08	27.31	0.90	0.33	1.48	2.25
	8.6 × 10^21^	38.52	0.79	0.73	1.03	1.51	33.13	0.83	0.71	1.10	1.56	29.73	0.84	0.69	1.15	1.62	34.45	0.85	0.66	1.21	1.70	31.62	0.87	0.62	1.25	1.76	25.54	0.92	0.50	1.37	1.95
	2.6 × 10^22^	31.18	0.90	0.76	0.96	1.30	30.17	0.92	0.79	0.96	1.25	31.73	0.92	0.80	0.97	1.25	30.35	0.92	0.80	0.99	1.27	30.62	0.92	0.80	0.99	1.27	36.19	0.90	0.78	1.02	1.34
	5.6 × 10^22^	33.18	0.90	0.77	0.95	1.28	41.12	0.90	0.80	0.94	1.24	50.94	0.89	0.82	0.98	1.27	36.82	0.86	0.83	1.03	1.34	47.62	0.85	0.83	1.04	1.36	55.16	0.83	0.82	1.04	1.39
	8.6 × 10^22^	34.35	0.90	0.78	0.94	1.26	46.88	0.89	0.81	0.95	1.25	61.80	0.87	0.82	1.00	1.31	40.08	0.83	0.83	1.06	1.40	57.07	0.81	0.83	1.07	1.43	65.96	0.79	0.83	1.07	1.45
	2.6 × 10^23^	36.24	0.89	0.78	0.94	1.27	56.41	0.87	0.81	0.95	1.27	81.68	0.83	0.83	1.03	1.37	45.23	0.77	0.84	1.11	1.50	73.81	0.74	0.84	1.13	1.55	86.67	0.73	0.83	1.13	1.57
	5.6 × 10^23^	36.81	0.89	0.78	0.94	1.27	59.38	0.86	0.81	0.96	1.29	88.47	0.82	0.83	1.04	1.39	46.77	0.75	0.84	1.12	1.53	79.35	0.73	0.83	1.14	1.58	94.06	0.71	0.83	1.15	1.61

**Table 3:** Same as Table 1, except for the viscosity variations of the transition zone versus D″ layers embedded within the optimal 1 model (the lithosphere viscosity of 2.6 × 10^22^ Pa-s and the asthenosphere viscosity of 14 × 10^19^ Pa-s within the viscosity structure SH08).

Items	Viscosities	Tomography models													
		S40RTS							SAW642AN						
	D″ Layers Transition zone	1.8 × 10^19^	5.8 × 10^19^	1.8 × 10^20^	5.8 × 10^20^	1.8 × 10^21^	5.8 × 10^21^	1.8 × 10^22^	1.8 × 10^19^	5.8 × 10^19^	1.8 × 10^20^	5.8 × 10^20^	1.8 × 10^21^	5.8 × 10^21^	1.8 × 10^22^
VRMS	5.5 × 10^18^	52.22	66.60	75.23	78.99	80.62	82.35	85.83	49.08	58.28	64.83	67.85	69.19	70.62	73.48
Geoid		0.53	0.41	0.37	0.33	0.32	0.31	0.30	0.59	0.45	0.41	0.35	0.34	0.33	0.32
GSRM		0.84	0.85	0.84	0.84	0.83	0.83	0.83	0.79	0.81	0.81	0.81	0.80	0.80	0.80
log RMS		1.09	0.97	0.94	0.92	0.93	0.93	0.93	1.22	1.05	0.99	0.95	0.94	0.95	0.95
Total errors		1.71	1.72	1.72	1.76	1.77	1.80	1.81	1.84	1.79	1.77	1.79	1.80	1.82	1.83
VRMS	1.5 × 10^19^	44.65	59.37	69.92	74.69	76.72	78.68	82.50	44.31	51.57	59.20	62.99	64.64	66.26	69.37
Geoid		0.67	0.50	0.45	0.38	0.37	0.33	0.32	0.73	0.56	0.50	0.42	0.41	0.36	0.35
GSRM		0.84	0.84	0.84	0.83	0.83	0.83	0.83	0.79	0.81	0.81	0.81	0.81	0.80	0.80
log RMS		1.12	1.00	0.96	0.94	0.94	0.94	0.94	1.25	1.08	1.02	0.95	0.95	0.95	0.95
Total errors		1.62	1.66	1.68	1.73	1.74	1.77	1.79	1.72	1.71	1.70	1.72	1.73	1.78	1.79
VRMS	5.5 × 10^19^	40.89	42.34	53.72	59.79	62.38	64.69	68.98	69.37	46.24	39.83	46.49	51.05	53.11	54.98
Geoid		0.85	0.70	0.62	0.49	0.48	0.45	0.43	0.89	0.76	0.68	0.55	0.53	0.50	0.48
GSRM		0.83	0.83	0.83	0.83	0.82	0.82	0.82	0.79	0.81	0.81	0.81	0.80	0.80	0.80
log RMS		1.13	1.01	0.98	0.94	0.94	0.95	0.95	1.24	1.08	1.02	0.96	0.96	0.96	0.96
Total errors		1.44	1.47	1.52	1.63	1.64	1.68	1.70	1.55	1.51	1.53	1.61	1.62	1.66	1.68
VRMS	1.5 × 10^20^	63.34	34.07	35.15	40.68	43.45	45.88	50.36	65.44	36.16	32.70	36.18	38.28	40.21	43.84
Geoid		0.84	0.82	0.75	0.59	0.57	0.53	0.51	0.91	0.86	0.80	0.65	0.63	0.59	0.57
GSRM		0.83	0.83	0.83	0.82	0.82	0.82	0.82	0.79	0.81	0.81	0.80	0.80	0.80	0.80
log RMS		1.11	1.01	0.97	0.95	0.95	0.95	0.95	1.22	1.07	1.01	0.96	0.96	0.96	0.96
Total errors		1.44	1.35	1.40	1.53	1.55	1.60	1.62	1.51	1.40	1.41	1.51	1.53	1.57	1.59
VRMS	5.5 × 10^20^	105.22	56.36	32.70	27.68	27.54	28.27	30.60	99.55	53.23	29.58	23.90	23.54	24.16	26.38
Geoid		0.65	0.76	0.81	0.84	0.84	0.81	0.80	0.81	0.88	0.91	0.90	0.90	0.87	0.85
GSRM		0.83	0.83	0.82	0.82	0.81	0.81	0.81	0.80	0.80	0.80	0.79	0.79	0.79	0.79
log RMS		1.07	0.99	0.96	0.95	0.95	0.95	0.95	1.15	1.03	0.99	0.96	0.96	0.97	0.97
Total errors		1.60	1.40	1.33	1.29	1.30	1.33	1.34	1.55	1.34	1.28	1.26	1.27	1.31	1.33
VRMS	1.5 × 10^21^	124.96	71.85	42.30	32.05	29.36	28.20	27.61	116.06	65.38	36.03	25.30	22.49	21.39	21.26
Geoid		0.62	0.71	0.76	0.83	0.83	0.83	0.82	0.79	0.85	0.88	0.91	0.91	0.90	0.89
GSRM		0.83	0.82	0.82	0.81	0.81	0.81	0.81	0.79	0.80	0.80	0.79	0.79	0.79	0.79
log RMS		1.07	0.99	0.96	0.95	0.95	0.96	0.96	1.14	1.02	0.99	0.96	0.96	0.97	0.97
Total errors		1.62	1.45	1.38	1.31	1.31	1.32	1.33	1.56	1.37	1.30	1.26	1.26	1.28	1.29
VRMS	5.5 × 10^21^	136.05	81.25	49.36	37.06	33.25	31.15	28.85	125.60	73.12	41.40	28.55	24.49	22.34	20.41
Geoid		0.58	0.65	0.69	0.77	0.78	0.79	0.80	0.76	0.81	0.84	0.90	0.90	0.91	0.91
GSRM		0.82	0.82	0.82	0.81	0.81	0.81	0.80	0.79	0.80	0.80	0.79	0.79	0.78	0.78
log RMS		1.06	0.99	0.97	0.95	0.96	0.96	0.96	1.12	1.02	0.98	0.97	0.97	0.97	0.97
Total errors		1.65	1.51	1.46	1.37	1.37	1.36	1.36	1.57	1.40	1.34	1.28	1.28	1.28	1.29
VRMS	1.5 × 10^22^	140.14	85.13	52.37	39.31	35.10	32.68	29.84	129.44	76.74	44.17	30.51	25.97	23.40	20.71
Geoid		0.58	0.65	0.69	0.76	0.77	0.79	0.79	0.75	0.81	0.84	0.89	0.90	0.90	0.91
GSRM		0.82	0.82	0.82	0.81	0.81	0.80	0.80	0.79	0.80	0.80	0.79	0.79	0.78	0.78
log RMS		1.06	0.99	0.97	0.96	0.96	0.96	0.96	1.12	1.02	0.98	0.97	0.97	0.97	0.98
Total errors		1.66	1.52	1.47	1.38	1.38	1.37	1.36	1.57	1.41	1.35	1.29	1.28	1.29	1.29
VRMS	5.5 × 10^22^	145.36	91.13	57.18	42.91	38.08	35.23	31.69	134.94	83.06	49.40	34.47	29.18	25.97	22.15
Geoid		0.58	0.64	0.67	0.75	0.76	0.78	0.79	0.75	0.80	0.82	0.88	0.89	0.90	0.90
GSRM		0.82	0.82	0.82	0.81	0.81	0.80	0.80	0.80	0.80	0.80	0.79	0.79	0.78	0.78
log RMS		1.05	0.99	0.97	0.96	0.96	0.96	0.96	1.10	1.01	0.99	0.97	0.97	0.98	0.98
Total errors		1.66	1.53	1.48	1.40	1.39	1.38	1.38	1.56	1.42	1.37	1.30	1.30	1.30	1.30

**Table 4:** Same as Table 1, except for the viscosity variations of the transition zone versus D″ layers embedded within the optimal 2 model (the lithosphere viscosity of 25 × 10^22^ Pa-s and the asthenosphere viscosity of 2 × 10^19^ Pa-s within the viscosity structure SH08).

Items	Viscosities	Tomography models													
		S40RTS							SAW642AN						
	D″ Layers Transition zone	1.8 × 10^19^	5.8 × 10^19^	1.8 × 10^20^	5.8 × 10^20^	1.8 × 10^21^	5.8 × 10^21^	1.8 × 10^22^	1.8 × 10^19^	5.8 × 10^19^	1.8 × 10^20^	5.8 × 10^20^	1.8 × 10^21^	5.8 × 10^21^	1.8 × 10^22^
VRMS	5.5 × 10^18^	113.81	109.96	107.88	107.06	107.02	107.76	109.88	93.47	90.06	88.15	87.38	87.31	87.88	89.54
Geoid		0.22	0.24	0.25	0.26	0.26	0.25	0.24	0.22	0.25	0.27	0.27	0.27	0.27	0.26
GSRM		0.83	0.81	0.80	0.80	0.79	0.79	0.79	0.80	0.79	0.79	0.78	0.78	0.78	0.77
log RMS		0.99	0.94	0.94	0.95	0.95	0.96	0.96	1.03	0.93	0.92	0.93	0.93	0.94	0.94
Total errors		1.94	1.89	1.89	1.89	1.90	1.92	1.93	2.01	1.89	1.86	1.88	1.88	1.89	1.91
VRMS	1.5 × 10^19^	96.29	98.84	99.92	100.35	100.76	101.77	104.26	79.84	81.24	81.76	81.98	82.27	83.05	84.98
Geoid		0.32	0.30	0.30	0.29	0.29	0.29	0.27	0.36	0.34	0.33	0.33	0.32	0.32	0.30
GSRM		0.82	0.81	0.80	0.79	0.79	0.79	0.78	0.80	0.79	0.78	0.78	0.78	0.77	0.77
log RMS		1.01	0.95	0.95	0.95	0.96	0.96	0.96	1.05	0.95	0.93	0.93	0.94	0.94	0.94
Total errors		1.87	1.84	1.85	1.87	1.88	1.88	1.91	1.89	1.82	1.82	1.82	1.84	1.85	1.87
VRMS	5.5 × 10^19^	53.31	65.11	72.87	76.29	77.79	79.44	82.83	52.07	57.17	62.07	64.46	65.56	66.81	69.38
Geoid		0.68	0.53	0.44	0.41	0.40	0.39	0.36	0.74	0.59	0.51	0.47	0.45	0.44	0.41
GSRM		0.82	0.81	0.80	0.79	0.79	0.79	0.79	0.80	0.79	0.78	0.78	0.78	0.77	0.77
log RMS		1.02	0.96	0.95	0.95	0.96	0.96	0.97	1.05	0.96	0.93	0.94	0.94	0.94	0.95
Total errors		1.52	1.62	1.71	1.75	1.77	1.78	1.82	1.51	1.58	1.64	1.69	1.71	1.73	1.77
VRMS	1.5 × 10^20^	54.84	37.72	42.65	47.47	49.72	51.79	55.73	60.11	41.08	41.17	43.91	45.42	46.90	49.78
Geoid		0.80	0.82	0.71	0.64	0.61	0.58	0.53	0.88	0.86	0.76	0.69	0.66	0.63	0.59
GSRM		0.82	0.81	0.80	0.80	0.79	0.79	0.79	0.80	0.79	0.79	0.78	0.78	0.78	0.77
log RMS		1.01	0.96	0.95	0.95	0.96	0.96	0.96	1.05	0.96	0.93	0.93	0.94	0.94	0.94
Total errors		1.39	1.33	1.44	1.51	1.56	1.59	1.64	1.37	1.31	1.38	1.46	1.50	1.53	1.58
VRMS	5.5 × 10^20^	108.79	60.75	37.75	31.99	31.15	31.24	32.37	103.06	58.50	36.91	31.32	30.41	30.41	31.30
Geoid		0.62	0.72	0.79	0.81	0.81	0.80	0.78	0.80	0.85	0.88	0.87	0.86	0.84	0.81
GSRM		0.82	0.81	0.81	0.80	0.80	0.80	0.79	0.79	0.79	0.79	0.78	0.78	0.78	0.78
log RMS		1.01	0.96	0.94	0.95	0.95	0.95	0.96	1.04	0.96	0.93	0.93	0.93	0.94	0.94
Total errors		1.57	1.43	1.34	1.34	1.34	1.35	1.39	1.45	1.32	1.26	1.28	1.29	1.32	1.35
VRMS	1.5 × 10^21^	133.20	79.96	50.46	39.72	36.50	34.76	32.81	123.28	73.16	45.07	34.89	31.94	30.46	29.04
Geoid		0.57	0.64	0.71	0.74	0.75	0.76	0.77	0.76	0.81	0.85	0.86	0.86	0.86	0.85
GSRM		0.82	0.81	0.81	0.80	0.80	0.80	0.79	0.79	0.79	0.79	0.78	0.78	0.78	0.78
log RMS		1.01	0.96	0.94	0.95	0.95	0.95	0.95	1.04	0.96	0.93	0.93	0.93	0.94	0.94
Total errors		1.62	1.51	1.42	1.41	1.40	1.39	1.39	1.49	1.36	1.29	1.29	1.29	1.30	1.31
VRMS	5.5 × 10^21^	146.32	91.16	59.14	46.53	42.37	39.89	36.57	134.52	82.24	51.45	39.28	35.34	33.09	30.36
Geoid		0.55	0.61	0.66	0.69	0.71	0.72	0.73	0.74	0.78	0.82	0.84	0.84	0.84	0.84
GSRM		0.82	0.81	0.81	0.80	0.80	0.80	0.80	0.79	0.79	0.79	0.78	0.78	0.78	0.78
log RMS		1.01	0.96	0.94	0.95	0.95	0.95	0.95	1.04	0.96	0.93	0.93	0.94	0.94	0.94
Total errors		1.64	1.54	1.47	1.46	1.44	1.43	1.42	1.51	1.39	1.32	1.31	1.32	1.32	1.32
VRMS	1.5 × 10^22^	151.02	95.59	62.62	49.27	44.77	42.05	38.31	138.92	86.32	54.56	41.61	37.29	34.75	31.52
Geoid		0.55	0.60	0.65	0.68	0.69	0.70	0.72	0.74	0.77	0.81	0.83	0.83	0.84	0.84
GSRM		0.82	0.81	0.81	0.80	0.80	0.80	0.80	0.79	0.79	0.79	0.79	0.78	0.78	0.78
log RMS		1.01	0.96	0.95	0.95	0.95	0.95	0.95	1.04	0.96	0.94	0.93	0.94	0.94	0.94
Total errors		1.64	1.55	1.49	1.47	1.46	1.45	1.43	1.51	1.40	1.34	1.31	1.33	1.32	1.32
VRMS	5.5 × 10^22^	156.97	102.25	67.89	53.28	48.19	45.10	40.79	145.17	93.28	60.19	45.94	40.96	37.94	33.97
Geoid		0.55	0.60	0.65	0.68	0.69	0.70	0.71	0.73	0.77	0.80	0.82	0.83	0.83	0.84
GSRM		0.82	0.81	0.81	0.80	0.80	0.80	0.80	0.79	0.79	0.79	0.79	0.78	0.78	0.78
log RMS		1.02	0.97	0.95	0.95	0.95	0.95	0.95	1.05	0.97	0.94	0.94	0.94	0.94	0.94
Total errors		1.65	1.56	1.49	1.47	1.46	1.45	1.44	1.53	1.41	1.35	1.33	1.33	1.33	1.32

We calculate the root mean square (hereby named VRMS in Table 1, Table 2, Table 3, Table 4) differences and the correlation coefficients (hereby named $Cor_{geoid}$) between the predicted and the observed geoid (VRMS and Geoid in Table 1, Table 2, Table 3, Table 4). We also compute the root mean square (mm/yr) differences between the calculated surface motions in an NNR frame with the velocities of Kreemer et al. (2006) [13], (hereby named $RMS_{velocity}$ and log RMS in Table 1, Table 2, Table 3, Table 4).

We define a total error: $\varepsilon_{total\_error}=1-Cor_{geoid}+1-Cor_{GSRM}+log\left(RMS_{velocity}\right)$ to show the effects of the viscosity variations of the lithosphere, asthenosphere, transition zone, and D″ layer easily, whose total errors are shown in Table 1, Table 2, Table 3, Table 4.
